# Myosin relaxation states in skeletal muscle fibers of rats and mice: Effects of sex and adiposity

**DOI:** 10.14814/phy2.70336

**Published:** 2025-04-13

**Authors:** Zachery A. Roloff, Lien A. Phung, Luke A. Weyrauch, Philip C. Woods, Shawna L. McMillin, Brian P. Sullivan, Rebecca Barok, Naixin Zhang, Katherine A. Murphy, Timothy D. O'Connell, Brendan J. Dougherty, David D. Thomas, Mark S. Miller, Dawn A. Lowe

**Affiliations:** ^1^ Department of Family Medicine and Community Health, Division of Physical Therapy and Rehabilitation Science University of Minnesota Minneapolis Minnesota USA; ^2^ Department of Biochemistry, Molecular Biology, and Biophysics University of Minnesota Minneapolis Minnesota USA; ^3^ Department of Kinesiology University of Massachusetts Amherst Massachusetts USA; ^4^ Department of Integrative Biology and Physiology University of Minnesota Minneapolis Minnesota USA

**Keywords:** adiposity, ATPase, myosin, myosin heavy chain, SRX

## Abstract

Myosin disordered‐ and super‐relaxed states (DRX and SRX, respectively) in skeletal muscle fibers are hypothesized to play key roles in thermogenesis and basal metabolic energy expenditure, raising potential for novel therapeutic targets for obesity and other metabolic diseases. Limited studies have investigated relationships between body composition or biological sex and myosin relaxed states. Using fluorescence‐based single‐nucleotide turnover, we report quantitative relationships of diet‐induced adiposity and sex with biochemical parameters of myosin relaxed states of rodent muscle fibers. Our main findings were: (1) adiposity had minimal to no effect on parameters of relaxed myosin states measured in fibers from rats and mice, (2) fibers from female rats and mice had 10%–20% shorter SRX lifetimes than those from males (*p* ≤ 0.035), (3) in rats, females had shorter DRX lifetimes than males, and (4) myosin heavy chain isoform had negligible impact on parameters of relaxed myosin states. We conclude that skeletal muscle energy utilization during rest, as measured by myosin ATPase, is affected minimally by adiposity, but differs by sex. Continued exploration of the metabolic implications of myosin transitioning between SRX and DRX will provide further understanding of muscle thermogenesis and whole‐body metabolism; in so doing, sex as a biological factor should be considered.

## Graphical abstract

Sex, and less‐so adiposity, affects the relaxed state of myosin in rodent skeletal muscle.

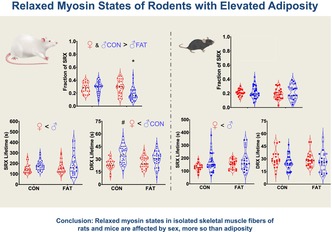

## 1 INTRODUCTION

Skeletal muscle comprises approximately 40% of total body mass and, during movement, expends substantial energy to fuel muscle contraction. Skeletal muscle also utilizes energy during rest, and given the tissue's large volume and low but essential resting energy expenditure, skeletal muscle is an important contributor to whole‐body metabolism (Zurlo et al., 1990). Emerging evidence highlights a pivotal role of relaxed (non‐contracting) skeletal muscle in metabolism through the specific kinetic states of the motor protein myosin. During muscle relaxation, myosin exhibits two biochemically distinct states: disordered‐relaxed (DRX) and super‐relaxed (SRX) states, which differ in ATP turnover rates (Stewart et al., 2010). Myosin heads in DRX are detached from the thick filament, oriented toward the thin filament, and lack actin interactions, resulting in a “disordered” configuration (Stewart et al., 2010). ATP turnover is rapid (<30 s) in the DRX state (McNamara et al., 2015; Stewart et al., 2010). Conversely, myosin heads in SRX fold back toward the thick filament backbone, causing auto‐inhibition of the catalytic domains on the myosin S1 fragment (Fusi et al., 2015; Woodhead et al., 2005), resulting in a more than five‐fold slower ATP turnover rate compared to DRX (McNamara et al., 2015). The physiological significance of myosin SRX is not fully understood, but the slow ATP turnover rate is theorized to contribute to the maintenance of low muscle thermogenesis and consequently low whole‐body metabolism relative to myosin in the DRX state (Stewart et al., 2010; Wilson et al., 2021).

Dynamic equilibrium exists between DRX and SRX myosin in non‐contracting skeletal muscle fibers, but at in vivo temperatures, it is theorized that the majority of myosin resides in SRX to conserve energy (Stewart et al., 2010; Wilson et al., 2021). It has been hypothesized that by selectively targeting SRX myosin and inducing a shift to DRX, muscle thermogenesis and whole‐body metabolism would increase and provide a foundation for potential therapeutic strategies. For example, calculations have been put forward to indicate that if all skeletal muscle SRX myosin were to transition to DRX, basal metabolic rate would increase by approximately 50%, equivalent to the expenditure of roughly 1000 kcal/day (Cooke, 2011). Such metabolic changes in skeletal muscle during rest could theoretically impact the management of obesity and obesity‐linked diseases such as metabolic syndrome and type II diabetes.

While studies of myosin in cardiac myocytes are revealing SRX‐DRX disease mechanisms (reviewed in (Solaro et al., 2024)), how relaxed myosin states impact skeletal muscle health and disease is less understood but is being increasingly studied. For example, Ranu and coworkers showed that in nemaline myopathy myosin SRX fraction is low and thus basal skeletal muscle ATP consumption in fibers from both rodents and humans is relatively high (Ranu et al., 2022). Early studies of skeletal muscle myosin relaxed states showed that decreased temperature (Stewart et al., 2010) or increased phosphorylation of myosin regulatory light chain (Naber et al., 2011; Stewart et al., 2010) destabilized SRX myosin and increased the fraction of myosin in DRX in rabbit fibers, providing some mechanistic insight into ATP turnover mechanics in relaxed skeletal muscle. Consistent with results of decreased temperature affecting relaxed myosin states, Lewis and coworkers showed that hibernation and cold exposure affected relaxed myosin states in skeletal muscle fibers from rodents and bears (Lewis et al., 2024).

Changes in SRX and DRX lifetimes have also been reported in response to ovariectomy (Colson et al., 2015) and aging in female but not male mouse skeletal muscle fibers (Phung et al., 2018). These adaptations to ATP turnover by relaxed myosin indicate a possible role of estrogen loss in the disruption of SRX myosin. However, a potential confounding variable in those studies (Colson et al., 2015; Phung et al., 2018) is that ovariectomy and aging in female mice typically result in increased body fat, which could have been a contributing factor to the measured differences in relaxed myosin states. To address the possibility that high body fat (~adiposity) affects ATP turnover by skeletal muscle myosin, here we used two different rodent models to administer specific chows that were high in dietary fat and have been successful at inducing adiposity (DeNies et al., 2014; Merat et al., 1999; Salmon & Flatt, 1985; Umek et al., 2021). We hypothesized that resting myosin ATP turnover would be low in conditions of adiposity due to fibers having relatively high SRX fraction (and low DRX fraction), and relatively long SRX and/or DRX lifetimes. We utilized the fluorescent nucleotide chase method to measure fractions and lifetimes of SRX and DRX in parallel studies of rat and mouse skeletal muscle fibers. Furthermore, our studies extended the scope to include sex‐based analyses and considered fiber types based on myosin heavy chain isoform expression. By measuring relaxed myosin states in fibers from adult female and male rodents with typical versus high body fat, we demonstrate that greater adiposity induces minimal alterations in SRX and DRX fractions and lifetimes, with notable distinctions between sexes, regardless of fiber type.

## 2 METHODS

### 2.1 Animals and experimental design (rats)

The first study was designed to determine if increased adiposity altered the parameters of relaxed skeletal muscle myosin, in a modest model of increased adiposity. ‘Adiposity’ is used as a general term for increased fat and is not in reference to fat in any particular part of the animal's body. Female (*n* = 11) and male (*n* = 10) Sprague–Dawley rats (RRID:RGD_734476) aged 6 months were fed normal chow (CON) or a Western‐style diet (FAT). The composition of the chow given to the CON groups was 58% carbohydrate, 18% fat, and 24% protein, while the chow for the FAT groups was 50% carbohydrate, 34.5% fat, and 15.5% protein (Teklad 2918 and TD.110424, Indianapolis, IN). Rats were housed in same‐sex pairs. Body composition was measured by EchoMRI (EchoMRI, Houston, TX) at the beginning of the study and after 12 week on the diet. The rats were then deeply anesthetized with isoflurane, euthanized via transcardial perfusion with ice‐cold saline (pH 7.4), and one soleus muscle per rat was dissected and used for measuring relaxed myosin properties. For this study, rat soleus muscle was selected for its unique characteristic of being entirely composed of fibers expressing myosin heavy chain type I (MHC I) (Bär & Pette, 1988), and muscles comprised of relatively more MHC I fibers accumulate a greater amount of intramuscular fat than those comprised of MHC II fibers (Komiya et al., 2017).

### 2.2 Animals and experimental design (mice)

A second study was conducted to determine if a more severe induction of adiposity altered parameters of the relaxed myosin states and also to consider fiber types as the mouse soleus muscle is composed of both MHC I and MHC II fibers. Female (*n* = 9) and male (*n* = 10) C57BL/6 mice (RRID:IMSR_JAX:000664) aged 3 months were a subset of those reported in Zhang et al. (2023) that were fed normal chow (CON) or a high‐fat, high‐sucrose diet (FAT). The chow for the CON groups consisted of 55.8% carbohydrate, 11% fat, 11% sucrose, 20.1% protein, and 2.1% vitamins and minerals (DYET #104607, Dyets Inc., Bethlehem, PA). The chow for the FAT groups was specifically designed to induce metabolic syndrome, which is a clustering of hypertension, type II diabetes, increased levels of plasma triglycerides, decreased plasma high‐density lipoprotein, and obesity (Huang, 2009; Panchal & Brown, 2011). The composition of this chow was 10.5% carbohydrate, 42% fat, 30% sucrose, 15.6% protein, and 1.9% vitamins and minerals (DYET #104608, Dyets Inc.). Mice were housed by sex in groups of 3–4. At the beginning of the study and after 20 week. on diet, body composition was measured using EchoMRI. Mice were deeply anesthetized with isoflurane and euthanized by ablation of the heart. One soleus muscle per mouse was dissected and used for measuring relaxed myosin properties, and contralateral soleus muscles were dissected and used in a triglyceride assay to provide insight into muscle adiposity induced by the diet.

All animals were maintained on a 12 h light/dark cycle and had access to their respective chow and water ad libitum. All protocols and animal care procedures were approved by the University of Minnesota Institutional Animal Care and Use Committee.

### 2.3 Fluorescent nucleotide chase experiments

Freshly dissected rat soleus muscles were cut in half longitudinally (parallel with fibers) and fiber bundle ends tied onto Drummond tubes with sutures. Whole mouse soleus muscle was dissected and tied onto Drummond tubes at both tendons. Rat fiber bundles and mouse soleus muscles had diameters of approximately 2 mm to ensure complete membrane permeabilization as previously described (Colson et al., 2015; Stewart et al., 2010). Samples were stored at −20°C for up to 3 months.

All solutions used in the chase experiments contained 120 mM K‐acetate, 5 mM K‐phosphate, 5 mM Mg‐acetate, 4 mM EGTA, and 50 mM MOPS, pH 6.8, with this base solution being referred to as rigor buffer (Stewart et al., 2010). Single fibers were manually isolated on the day of experiments under relaxed conditions (rigor buffer plus 4 mM ATP) using an Olympus SZX‐ILLK100 microscope (Olympus, Center Valley, PA). Fibers were either mounted on 35 mm glass bottom culture dishes or 30 mm coverslips that were used with an interchangeable cover‐glass dish (Bioptechs, Butler, PA). Fibers were held in place with vacuum grease on both ends.

**FIGURE 1:**
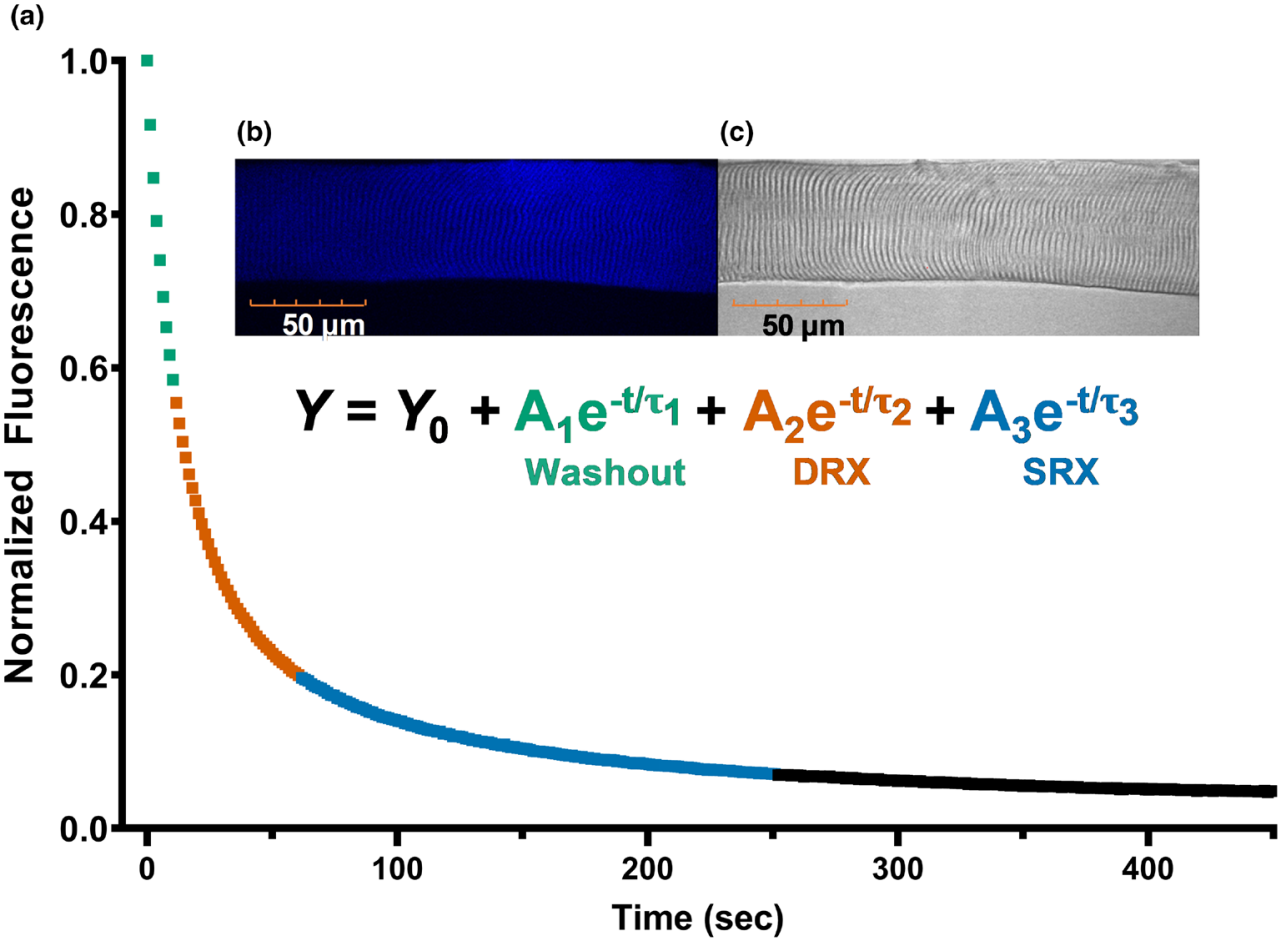
Measuring fluorescence decay of a rodent skeletal muscle fiber during a chase experiment. (a) Representative normalized fluorescence signals of a rodent muscle fiber during the fluorescent nucleotide exchange process, fitted with a 3‐exponential decay function. The first decay term (green) is the nonspecific washout period. The second and third exponential terms represent the DRX and SRX, respectively. Inset is a segment of an isolated single fiber, loaded with mant‐ATP, imaged under (b) fluorescence and (c) bright‐field.

Ambient temperature was recorded each experimental day and averaged 23.0 ± 0.7°C. The temperature of the solutions used was measured using a microprobe thermometer and averaged 22.2 ± 1.4°C. ATP chase experiments were conducted on an Olympus FV1000 IX2 confocal microscope with a 60x UPlanApo N oil immersion objective lens (1.42 NA) for both differential interference contrast and fluorescence imaging. All images for chase experiments were scanned in a 512 × 512‐pixel grid with a total exposure time of 1.1 s, and an effective pixel size of 414 nm. Fibers were initially incubated for 5 min in rigor buffer containing 250 μM 2′−/3′‐O‐(N′‐Methylanthraniloy)‐adenosine‐5′‐O‐triphosphate (mantATP, #M12417; ThermoFisher Scientific, Waltham, MA), and the fluorescent signal was confirmed to be stable with 405 nm excitation and 475/50‐nm emission wavelength filters. After the incubation period, the solution was exchanged with rigor buffer containing 4 mM ATP. The fluorescent decay signal was tracked over 450 s starting when the 4 mM ATP solution was added to the fiber. The signal was analyzed as the intensity of the fiber minus the intensity of the background in the same image, then normalized to the peak intensity value at the start of each chase. Data were fit to a 3‐exponential decay function using a nonlinear least‐squares algorithm in Origin 2023 (RRID:SCR_014212, OriginLab Corp., Northampton, MA) (Figure 1). Previous studies have used both 2‐ and 3‐exponential decay functions, but comparisons from Phung et al. demonstrated a better fit when a third term is added to the formula (Phung et al., 2018). Upon completion of the chase experiment, each fiber was collected and analyzed for MHC isoform content via SDS‐PAGE as described (Miller et al., 2010; Phung et al., 2020), with minor modifications to the resolving gel (8% acrylamide/bis‐30% glycerol (w/v)) and running conditions (70 V for 1 h, then 275 V for 26 h).

### 2.4 Skeletal muscle triglyceride content

A triglyceride assay was used to further characterize the different responses to the fat‐inducing condition in mice. Soleus muscles were homogenized using a glass‐on‐glass tissue homogenizer and lipids were extracted using a 2:1 chloroform: methanol solution. After separation, the chloroform phase was removed and evaporated with N_2_, then diluted with 1% Triton X‐100 in isopropanol. Samples were placed in a 96‐well plate along with Infinity reagent (#TR22421; ThermoFisher) and read on a SpectraMax iD3 plate reader (Molecular Devices, San Jose, CA) to measure total triglyceride concentration.

### 2.5 Statistical analyses

Levene's test was used to determine homogeneity of the variance assumption, and the Shapiro–Wilk test was conducted to investigate within‐group normality of each parameter. Overall, 265 fibers were analyzed and 15 were removed as outliers based on robust regression and outlier detection (ROUT) test results. An a priori power analysis was conducted to determine the necessary sample size to detect medium effect sizes with a significance level of *α* = 0.05 and power of 0.80. Two‐way ANOVAs with Holm‐Sidak post hoc tests were used to analyze body and soleus muscle compositions. Myosin relaxed‐state fractions and lifetimes between sexes and between control (CON) and fat‐inducing (FAT) conditions were analyzed using a linear mixed model, including a random effect to account for clustering of observations within animals. Correlations were carried out using Pearson's correlation coefficient, and ANCOVAs were used to analyze differences between regression lines. *p*‐values less than 0.05 were considered statistically significant. Statistical analyses were performed using commercially available statistical analysis software (RRID:SCR_000432, R ver. 4.3.3, R Core Team, Vienna, Austria; RRID:SCR_002798, Prism ver. 8, GraphPad, La Jolla, CA). All data are presented as mean ± SD.

## 3 RESULTS

### 3.1 Rat soleus muscle

#### 3.1.1 Body and muscle characteristics

Rat body and muscle characteristics are shown in Table 1. Body mass and body fat percent were significantly greater in the FAT groups compared to CON groups, indicating that the fat‐inducing condition was successful at increasing adiposity in both female and male rats. The fat‐inducing condition did not affect soleus muscle mass. As expected, there was a main effect of sex on body and soleus muscle masses, with males being greater. There was no significant effect of sex on body fat percentage.

**TABLE 1:** Body and soleus muscle characteristics of female and male rats fed normal chow (CON) or adiposity‐inducing chow (FAT) in Study 1.

	Female		Male		Two‐way ANOVA *p*‐value		
	CON	FAT	CON	FAT	Condition	Sex	Sex × Condition
*n*	5	6	5	5			
Body Mass (g)	240.0 ± 18.8	299.7 ± 23.2	381.8 ± 19.1	423.0 ± 20.9	<0.001	<0.001	0.357
Body Fat (%)	9.8 ± 1.7	12.9 ± 2.6	10.1 ± 1.2	11.9 ± 1.1	0.020	0.690	0.482
Soleus Mass (mg)	73.1 ± 11.2	85.5 ± 13.1	123.7 ± 18.7	125.0 ± 29.0	0.292	0.032	0.657
Soleus: Body Mass (mg/g)	0.30 ± 0.03	0.28 ± 0.03	0.12 ± 0.02	0.14 ± 0.02	0.257	0.598	0.993

*Note*: Data are mean ± SD.

#### 3.1.2 Myosin relaxed state fractions and lifetimes in rat skeletal muscle fibers

**FIGURE 2:**
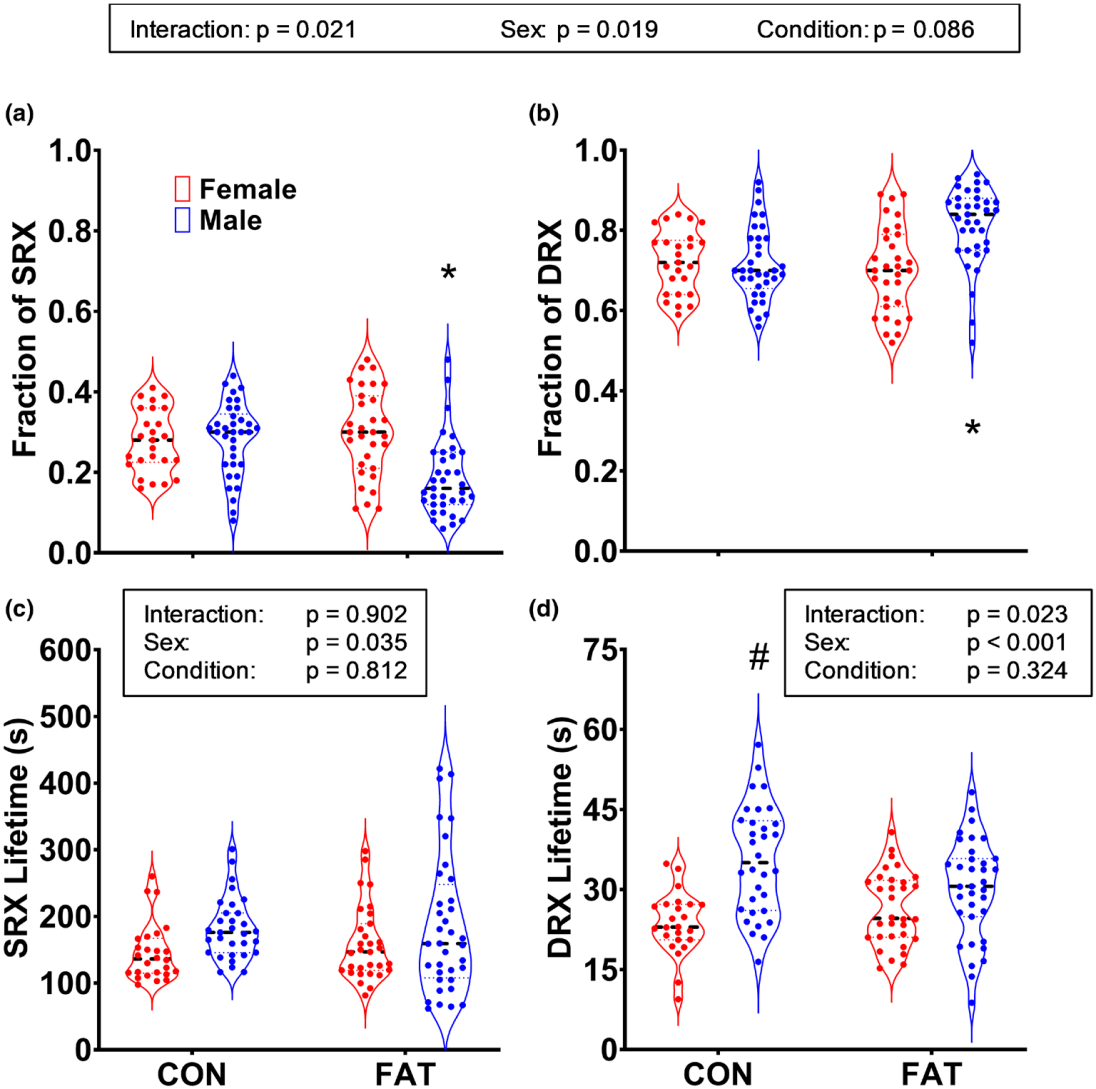
Relaxed skeletal muscle myosin fractions (a, b) and lifetimes (c, d) in male and female rats fed normal chow (CON) or a chow to induce adiposity (FAT). Each data point represents an individual fiber (*n* = 27–36 fibers per each of four groups with *n* = 4–9 fibers per rat soleus muscle). *Significantly different from all other groups via post hoc analyses (*p* ≤ 0.027); ^#^Significantly different from female groups via post hoc analyses (*p* < 0.001).

Analysis of the SRX and DRX fraction data revealed a main effect of condition, but only in the males. Fibers from the male FAT group had a ~30%–36% smaller fraction of SRX myosin (and greater DRX) than that of fibers from all other groups (Figure 2a,b). SRX lifetime was ~20% shorter in fibers from female rats compared to males and was not affected by condition (Figure 2c). DRX lifetime was longer in fibers from male CON rats than in all other groups (Figure 2d).

### 3.2 Mouse soleus muscle

#### 3.2.1 Body and muscle characteristics

Mouse body and muscle characteristics are shown in Table 2. The fat‐inducing condition resulted in significantly greater body mass and body fat percent compared to the CON groups. As expected, body and soleus muscle masses were less in females compared to males. There were trends toward greater soleus muscle mass (*p* = 0.072) and triglyceride content (*p* = 0.078) in the FAT mice compared to the CON mice. Overall, the results indicate that the fat‐inducing condition was successful at increasing adiposity in both the FAT female and male mice.

**TABLE 2:** Body and soleus muscle characteristics of female and male mice fed normal chow (CON) or adiposity‐inducing chow (FAT) in Study 2.

	Female		Male		Two‐way ANOVA *p*‐value		
	CON	FAT	CON	FAT	Condition	Sex	Sex × condition
*n*	4	5	6	6			
Body Mass (g)	30.0 ± 3.6	44.1 ± 4.7	39.0 ± 5.5	50.8 ± 3.9	<0.001	0.003	0.604
Body Fat (%)	26.7 ± 8.1	50.4 ± 8.9	24.9 ± 13.1	37.1 ± 1.3	<0.001	0.101	0.206
Soleus Mass (mg)	9.7 ± 1.4	9.9 ± 1.3	10.2 ± 1.1	13.1 ± 2.4	0.072	0.039	0.113
Soleus: Body Mass (mg/g)	0.32 ± 0.03	0.23 ± 0.04	0.26 ± 0.03	0.26 ± 0.03	0.007	0.398	0.016
Soleus Triglyceride	1.00 ± 0.4	1.20 ± 0.6	1.00 ± 0.3	1.57 ± 0.4	0.078	0.386	0.382

*Note*: Soleus muscle triglyceride content normalized to control condition within sex. Data are mean ± SD.

#### 3.2.2 Myosin relaxed‐state fractions and lifetimes in mouse skeletal muscle fibers

**FIGURE 3:**
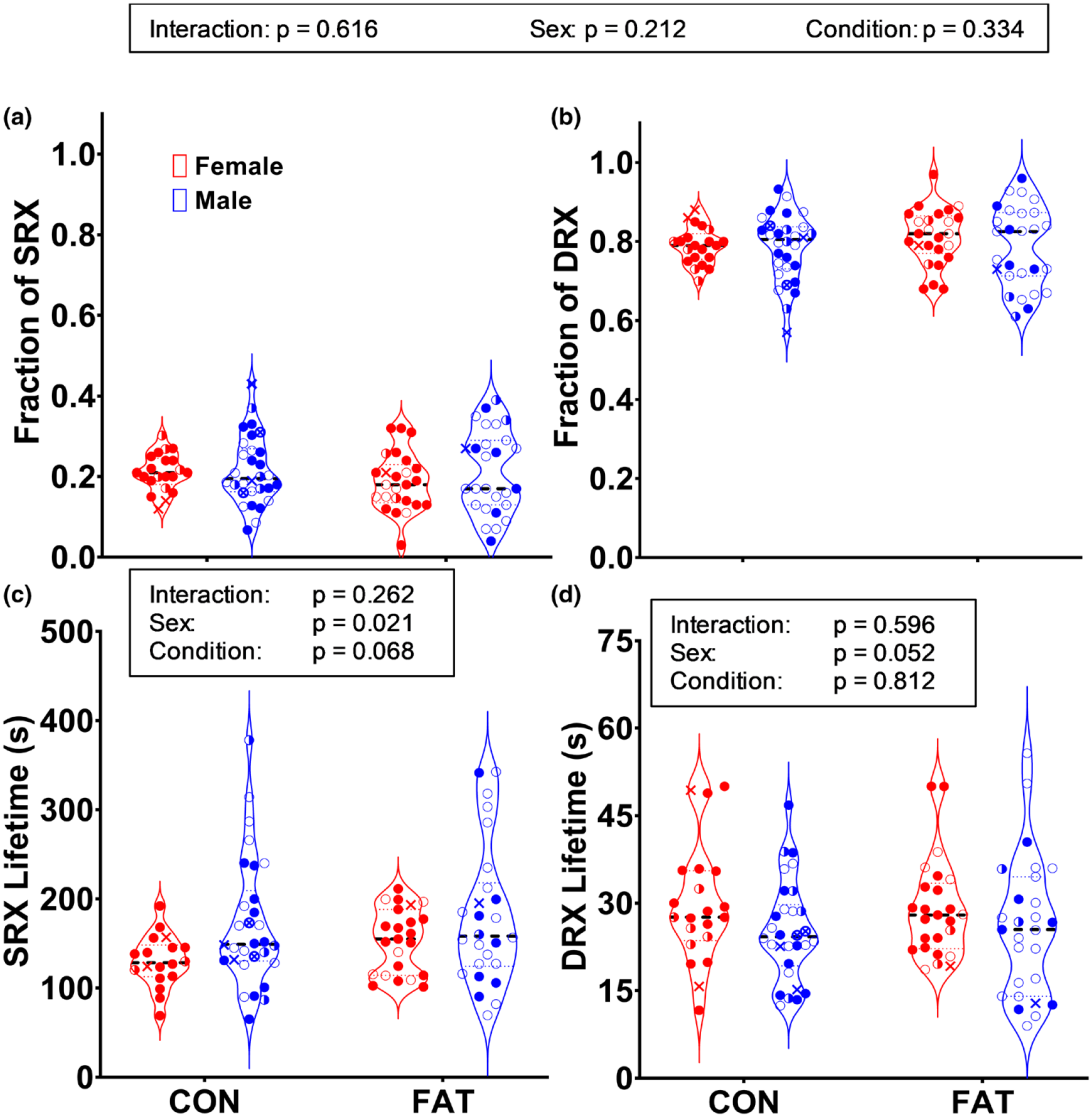
Relaxed skeletal muscle myosin fractions (a, b) and lifetimes (c, d) in male and female mice fed normal chow (CON) or a chow to induce adiposity (FAT). Each data point represents an individual fiber (*n* = 18–30 fibers per each of four groups with *n* = 4–8 fibers per mouse soleus muscle). Statistical results presented in boxes are those for all fibers collapsed across MHC fiber type. Filled circle = MHC I; open circle = MHC IIA; X = MHC IIX; half‐filled circle = MHC I/IIA; open circle with X = MHC IIAX.

Myosin relaxed‐state data from fibers of mice were first analyzed irrespective of fiber type, which revealed no effects of the fat‐inducing condition on fractions of SRX and DRX myosin (Figure 3a,b). However, fibers from females had significantly shorter SRX lifetimes than fibers from males (145 ± 41 vs. 169 ± 70 s, respectively; Figure 3c). There was a trend toward longer SRX lifetimes in the FAT compared to CON mice (*p* = 0.068), and a trend toward longer DRX lifetimes in fibers from females compared to males (*p* = 0.052; Figure 3d).

Mouse soleus muscle is composed of fibers containing multiple MHC isoforms; therefore, we next assessed myosin relaxed‐state parameters within fiber types (visually differentiated by symbols within Figure 3, statistical analyses in Table 3). For MHC I fibers, there was no effect of condition or sex on any relaxed myosin property. One‐way ANOVA was performed for MHC IIA fibers because, within the female CON group, none of the fibers analyzed were typed as IIA. There were no differences detected among the three groups for fraction or lifetime of SRX or DRX in MHC IIA fibers. Due to the low number of MHC IIX (*n* = 6) and hybrid fibers (*n* = 15), no statistical analyses were completed on those fiber types.

**TABLE 3:** Relaxed myosin state parameters for MHCI and IIA fibers from female and male mice under conditions of normal chow (CON) or adiposity‐inducing chow (FAT).

MHC I fibers	Female		Male		*p*‐value (2‐way ANOVA)		
	CON (*n* = 13)	FAT (*n* = 17)	CON (*n* = 10)	FAT (*n* = 6)	Condition	Sex	Sex × condition
SRX Fraction	0.21 ± 0.03	0.19 ± 0.1	0.20 ± 0.1	0.20 ± 0.1	0.515	0.789	0.759
SRX Lifetime (s)	177 ± 118	178 ± 82	155 ± 57	179 ± 82	0.135	0.132	0.924
DRX Lifetime (s)	29 ± 9	31 ± 10	25 ± 11	25 ± 11	0.329	0.070	0.404

MHC IIA Fibers	Female		Male		*p*‐value (1‐way ANOVA)		
	CON (*n* = 0)	FAT (*n* = 6)	CON (*n* = 13)	FAT (*n* = 18)	Main Effect		
SRX Fraction	n/a	0.16 ± 0.03	0.19 ± 0.07	0.20 ± 0.09	0.640		
SRX Lifetime (s)	n/a	170 ± 35	190 ± 69	214 ± 150	0.684		
DRX Lifetime (s)	n/a	29 ± 8	26 ± 7	26 ± 13	0.751		

*Note*: Data are mean ± SD.

To determine if MHC I and MHC IIA fibers differed in their relaxed myosin state properties, we collapsed the data across condition and sex. No significant differences between MHC I and IIA fibers were detected in SRX fractions (0.20 ± 0.08 vs. 0.19 ± 0.08, respectively; *p* = 0.381), SRX lifetimes (155 ± 60 vs. 176 ± 69 s, respectively; *p* = 0.141) or DRX lifetimes (29 ± 10 vs. 26 ± 10 s, respectively; *p* = 0.220).

## 4 DISCUSSION

A theory has been proposed that excessive caloric intake can lead to an increase in muscle thermogenesis that is in part due to altered ATP turnover kinetics by myosin in non‐contracting skeletal muscle (Stewart et al., 2010). Here we directly tested the hypothesis that diet‐induced adiposity affects fractions and lifetimes of myosin biochemical SRX and DRX states in skeletal muscle fibers from female and male rodents. Our main findings were that (1) adiposity had minimal to no effect on the parameters of the relaxed states of myosin measured in rats or mice, (2) fibers from female rats and mice had shorter SRX lifetimes than those from males, (3) in rats, females had shorter DRX lifetimes than males, and (4) MHC fiber type had little impact on myosin relaxed states.

### 4.1 Effect of adiposity on relaxed myosin states

In rats and mice, the fat‐inducing condition did not lead to significant effects on the fractions or lifetimes of myosin SRX or DRX. These data indicate that neither the local muscle environment of elevated intramuscular fat, as shown by relatively high muscle triglyceride content in mice fed the high‐fat diet, nor the systemic environment demonstrated by increased body fat composition of rats and mice fed high‐fat diets, affects myosin ATPase properties in non‐contracting muscle. Our results on fibers from rodents are in alignment with human muscle biopsy work done by Wilson et al. (2021) that failed to show significant relationships between relaxed myosin parameters and body mass index, which is a widely used yet simplistic estimate of body fat in humans. Additional caveats of the two studies include consideration of the differences in adipose tissue localization between rodents and humans, as well as between females and males, the relatively small numbers of subjects in the studies, and the lack of robust measures of intramuscular fat. Future studies on the topic of myosin relaxed states and adiposity building off initial results here will be strengthened by the inclusion of direct measures of muscle and fiber level adiposity as well as metabolic outcomes. While our results did not support the hypothesis that diet‐induced adiposity affects myosin relaxation states, we show evidence of sex‐specific differences in myosin SRX fraction and lifetime in fibers from both rats and mice.

### 4.2 Effect of sex on relaxed myosin states

Notable distinctions between sexes were measured in myosin SRX and DRX parameters, irrespective of adiposity conditions. Fibers from females of both rats and mice differed from males in utilization of ATP by myosin in SRX as lifetime was ~15% shorter in females, while in rats DRX lifetime was also shorter in females by ~30%. Our results indicating that males utilize ATP to a lesser extent in non‐contracting muscle than females are not consistent with previous research suggesting that low estrogen levels are partially responsible for destabilizing the SRX (Colson et al., 2015; Phung et al., 2018). Colson et al. measured shorter SRX lifetimes in fibers from adult ovariectomized mice (i.e., low estrogen state) compared to those from ovary‐intact mice. Notably, estradiol treatment to ovariectomized mice reversed the changes in SRX lifetime (Colson et al., 2015). Another study that used an aging model of estrogen deficiency showed that fibers from aged, ovarian‐senescent female mice also had shorter SRX and DRX lifetimes, with a trend toward a lower SRX fraction compared to their adult female counterparts, but no difference in any relaxed myosin parameter was detected between adult and aged male mice (Phung et al., 2018). Together, these two studies show that skeletal muscle fibers from estrogen‐deficient female mice have a destabilized myosin SRX state with faster relaxed myosin ATPases. However, our results here demonstrate that the relatively low estrogen state of the male sex relates to longer, not shorter, SRX and DRX lifetimes compared to the estrogenic state of the female sex.

With these conflicting results, mechanisms other than adiposity, and in addition to estrogen deficiency, appear to be involved in modulating relaxed myosin states differentially by sex. A protein that has been shown to be implicated in relaxed myosin states is myosin‐binding protein‐C (MyBP‐C). MyBP‐C is a thick filament‐associated protein important for the structure and function of striated muscle, including a role in muscle relaxation and stabilizing the SRX state in cardiac muscle, contributing to the regulation of myosin head activity (Nelson et al., n.d.; McNamara & Sadayappan, 2018). MyBP‐C is localized to the C‐zone of the sarcomere, where the majority of super‐relaxed myosin resides (Nelson et al., 2020). Mutations to cardiac MyBP‐C limit the inhibition of cross‐bridge cycling (McNally et al., 2015) and destabilize the SRX, resulting in less myosin in SRX and shorter SRX lifetime (Nelson et al., n.d.; McNamara et al., 2016, 2019). There are clinical phenotypic sex differences in hypertrophic cardiomyopathy resulting from mutations in cardiac MyBP‐C. Women with a MyBP‐C mutation have a later onset of disease but more frequent heart failure events compared to men, and women with hypertrophic cardiomyopathy are more likely to have sarcomere mutations (Lakdawala et al., 2021; Terauchi et al., 2015). In skeletal muscle, there are two paralogs of MyBP‐C, one of fast‐twitch muscle (fsMyBP‐C) and one of slow‐twitch muscle (ssMyBP‐C) (McNamara & Sadayappan, 2018). Both types of skeletal muscle MyBP‐C exhibit properties similar to cardiac, making it plausible that MyBP‐C may have a similar sex‐specific role in the modulation of skeletal muscle myosin relaxed states.

### 4.3 Myosin relaxation states with respect to fiber type and ATP consumption

It is well known that myosin ATPase during contraction greatly differs between MHC fiber types; thus, it is important to also consider the influence of MHC fiber type on myosin ATPase during relaxation. When all mouse soleus muscle fibers were analyzed together (i.e., disregarding fiber type) there were no significant main effects of sex or adiposity condition, with the exception of a sex difference in SRX lifetime in rats. The results of sex or adiposity condition on relaxed myosin properties, when MHC I and IIA fibers were analyzed separately, were similar to when analyzed together. We also show that the fractions and lifetimes of SRX and DRX myosin do not differ between MHC I and IIA fibers from mouse soleus muscle. Other studies have shown that the biochemical SRX and DRX states of myosin are dependent on MHC fiber types. One study in mice examined how muscle disease altered the SRX and DRX parameters (Laitila et al., 2024), and a human study investigated changes in the relaxed myosin parameters following a chronic physical activity intervention (Lewis et al., 2023). Additionally, ATP consumption during non‐contraction has been calculated based on relaxed myosin properties, and conditions such as fiber type and hibernation can affect the amount of ATP consumed per fiber per minute (Lewis et al., 2024). Using this calculation, our data show a ~20% greater ATP consumption in fibers from female rats compared to males, but there were no differences in mouse fibers or in either animal in response to adiposity. These results further emphasize the importance of sex differences and the potential for fiber types to play a role in ATP dynamics under varying physiological conditions.

In conclusion, our studies provide initial data to test theorized links between adiposity and relaxed myosin, and mark direct sex differences with respect to the biochemical SRX and DRX states of myosin. Fibers from female rodents had ~10%–20% faster ATP turnover by myosin in SRX compared to those from males, regardless of the adiposity of the rodent from which the fibers were harvested. The sex‐specific alterations in myosin relaxed‐state parameters unrelated to adiposity suggest that body fat may not act as a strong modulator of myosin relaxed states, and that sex is more influential on the equilibrium between SRX and DRX. The novel finding of the sex difference in myosin relaxed states is a reminder that research on skeletal muscle‐related therapeutic management of muscle and metabolic diseases should consider biological sex as a critical variable.

## AUTHOR CONTRIBUTIONS

ZAR, LAP, LAW, SLM, BPS, MSM, and DAL conceived and designed research; ZAR, LAW, PCW, BPS, SLM, RB, NZ, and KAM performed experiments; ZAR and PCW analyzed data; ZAR, LAP, LAW, PCW, SLM, DDT, MSM, and DAL interpreted results of experiments; ZAR prepared figures; ZAR and DAL drafted manuscript; All authors edited and revised manuscript, and all authors approved the final version of the manuscript.

## FUNDING INFORMATION

This work was supported by National Institutes of Health Grants (T32‐AR007612, R01‐AG031743, R01‐HL146477, R01‐HL152215, and R01‐AR082533).

## CONFLICT OF INTEREST STATEMENT

No conflicts of interest, financial or otherwise, are declared by the authors.

## ETHICS STATEMENT

All experiments involving animals were reviewed and approved by the Institutional Animal Care and Use Committees at the University of Minnesota and were in accordance with guidelines outlined by the Physiological Society.

## Data Availability

All data supporting the results reported above are stored on a secure computer at the University of Minnesota. Data are available from the senior author upon reasonable request.
